# Late-onset postoperative *Mycobacterium haemophilum* endophthalmitis masquerading as inflammatory uveitis: a case report

**DOI:** 10.1186/s12879-018-2985-0

**Published:** 2018-02-07

**Authors:** Warinyupa Pinitpuwadol, Sucheera Sarunket, Sutasinee Boonsopon, Nattaporn Tesavibul, Pitipol Choopong

**Affiliations:** Department of Ophthalmology, Faculty of Medicine, Siriraj Hospital, Mahidol University, 2 Wanglang Road, Bangkok Noi, Bangkok, 10700 Thailand

**Keywords:** Atypical mycobacteria, Non-tuberculous mycobacteria, Endophthalmitis, *Mycobacterium haemophilum*

## Abstract

**Background:**

Although atypical mycobacteria had been increasingly found in various ocular infections in the past decades, a slow-growing *Mycobacterium haemophilum (M. haemophilum)* was scarcely reported. Similar to tuberculous infection, the presentation can masquerade as low-grade granulomatous intraocular inflammation with partial response to corticosteroids. Besides, the special requirements for culture make this pathogen difficult to diagnose. The study aims to report the clinical presentation and notify the awareness of NTM endophthalmitis among clinicians. This is the first case report of late-onset, postoperative *M. haemophilum* endophthalmitis in the literature.

**Case presentation:**

A 66-year-old man with non-insulin-dependent diabetes mellitus (NIDDM) manifested chronic granulomatous inflammation in the left eye after multiple glaucoma surgeries. With a diagnosis of noninfectious panuveitis, he was treated with systemic corticosteroids. The inflammation initially responded to therapy although it subsequently worsened and became purulent endophthalmitis. The vitreous cultures grew *M. haemophilum*. Intraocular and systemic antimicrobial treatments were administered early, but the patient eventually turned blind.

**Conclusions:**

*M. haemophilum* endophthalmitis is a rare but serious intraocular complication leading to loss of vision or eyeball. Awareness of atypical mycobacterial infections is necessary especially in patients with impaired immune function, previous intraocular surgery, and corticosteroid resistance. Proper laboratory investigations and treatments should be performed. However, due to the rarity of the disease, the development of guidelines for its investigation and therapy is still challenging.

## Background

Non-tuberculous mycobacteria (NTM), or atypical mycobacteria, are described as *Mycobacterium species* other than *M. tuberculosis.* One of these, *M. haemophilum,* is a member of the slow-growing NTM which is non-photochromogenic and grows over two-four weeks [1]. This aerobic, non-spore-forming, gram-positive bacterium is found in the general environment; some studies have suggested that water is the main reservoir of *M. haemophilum* [2–5].

*M. haemophilum* was first introduced as a new pathogen in 1978 in a case of Hodgkin’s lymphoma presenting with chronic skin infection [6]. Later, it was reported in a variety of infections, mainly in immunocompromised hosts and children [3, 4, 7–9]. Three cases of *M. haemophilum* ocular infections have been reported in literature. Millar et al. reported an acute myeloid leukemia patient with NIDDM who developed chronic conjunctivitis and interstitial keratitis with multiple skin lesions on the face and arms [10]. Zuercher et al. reported a case of healthy child who presented with fistulous dacryocystitis and multiple cervical lymphadenopathies [11]. The only intraocular *M. haemophilum* infection was reported by Modi et al. in 2007; the authors described a case of chronic endogenous endophthalmitis with multiple skin nodules in a patient who was receiving immunosuppressive drugs after cardiac transplantation [12].

In this paper, we report a case of late-onset, postoperative endophthalmitis caused by *M. haemophilum* at the Department of Ophthalmology, Siriraj Hospital, Mahidol University, Bangkok, Thailand. Our patient manifested with an inflammatory uveitis-like presentation which partially responded to systemic corticosteroids and later became infectious endophthalmitis. He ended up with phthisis bulbi. To our knowledge, this is the first case of late-onset, postoperative endophthalmitis caused by *M. haemophilum*.

## Case presentation

A 66-year-old Thai man with bilateral advanced primary open angle glaucoma (POAG) presented to our clinic with chronic panuveitis for four months. His medical conditions included hypertension, hypercholesterolemia, and non-insulin-dependent diabetes mellitus (NIDDM) with an HbA1C of 8.2%. The patient had undergone three trabeculectomy surgeries in each eye; the last surgery having been performed in the left eye five years prior to the intraocular inflammation. Four months before presentation to our clinic, he complained of diminution of vision, pain, and redness in the left eye, which was treated as inflammatory panuveitis by an ophthalmologist at a local hospital. The treatment included topical and systemic corticosteroids. The inflammation improved during the three-month treatment period, but recurred after discontinuation of the medications. The second attempt showed minimal improvement over one month. Therefore, he stopped the medications and sought our opinion.

**Fig. 1:**
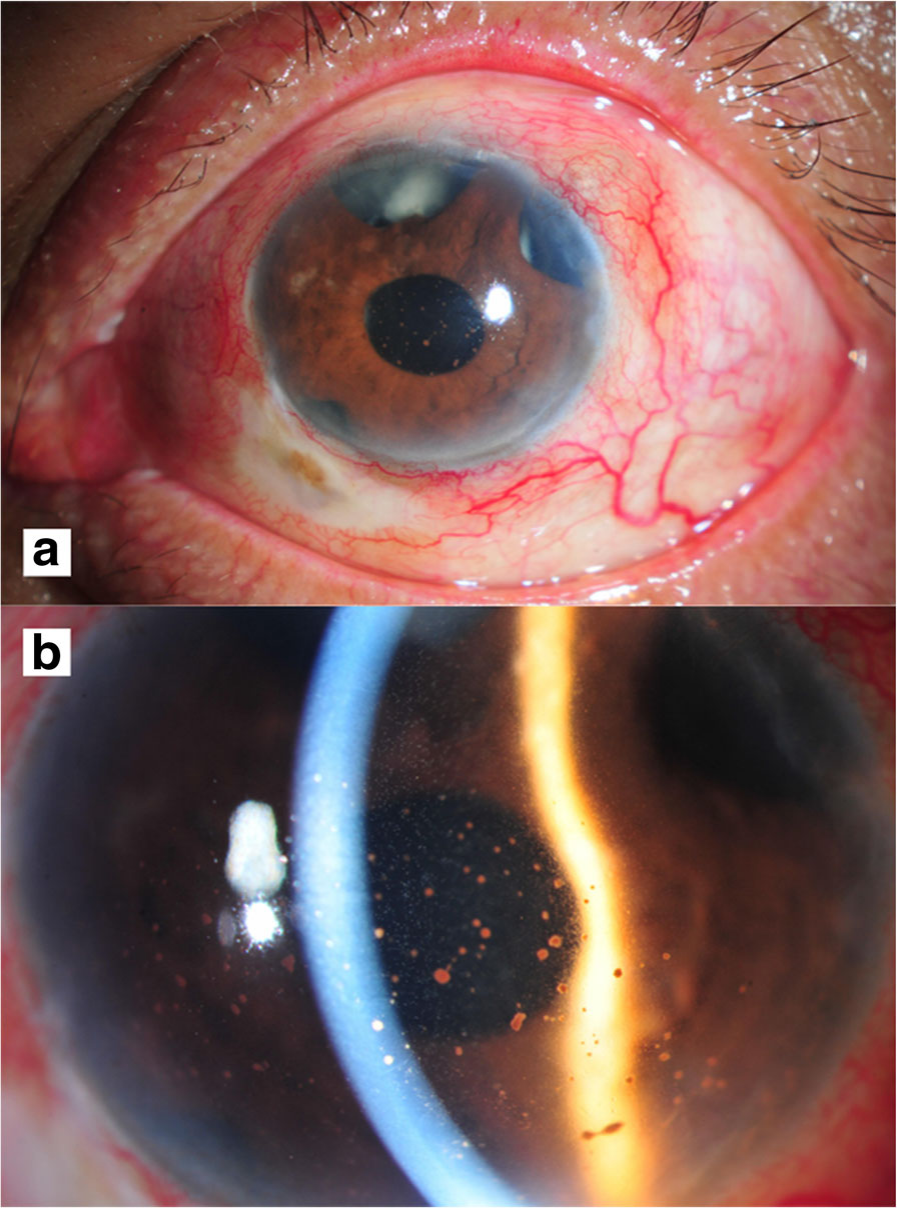
Slit lamp examination of the left eye at initial presentation. **a** Three flat trabeculectomy blebs with moderate circumcorneal conjunctival injection (**b**) Plasmoid aqueous, 4+ cells, and several large mutton fat keratic precipitates in the anterior chamber

At our service, the patient reported blurred vision with no ocular pain. The initial examination showed 6/6 best-corrected visual acuity (BCVA) in the right eye, and a poor response to light projection (PJ) in the left eye. The intraocular pressure (IOP) measurements were 21 mmHg in the right eye and 15 mmHg in the left eye. Slit-lamp examination of the left eye showed three flat trabeculectomy blebs with moderate circumcorneal conjunctival injection without discharge. Several large, mutton-fat keratic precipitates (KPs) were observed. There were plasmoid aqueous and 4+ cells in the anterior chamber (Fig. 1). A fundus examination revealed severe vitritis with a string-of-pearls appearance. The right eye was normal with three flat trabeculectomy blebs.

A moderate heterogenous vitreous echogenicity and attached retina appeared on B-scan ultrasonography. Chest radiography was normal. Laboratory results for anti-HIV, VDRL, TPHA, rheumatoid factor, antinuclear antibodies, and toxoplasma serology were all negative. Unfortunately, tests for serum lysozyme and angiotensin-converting enzyme were not performed as they were not available in Thailand. A presumptive diagnosis of severe panuveitis was made although infectious causes could not be excluded. Oral prednisolone was started at a dosage of 1 mg/kg/day with caution. The inflammation subsided. The visual acuity improved to hand motion (HM), and the prednisolone was gradually tapered.

One month later, his symptoms worsened with moderate ocular pain. Intense inflammation, including 4+ cells, iris fibrinous membrane, and hypopyon, was observed in the left eye. The inferior filtering bleb became inflamed. Due to these findings, infectious endophthalmitis became more suspicious. Aqueous and vitreous aspirations for cultures and molecular detections were performed to identify the organism, along with intravitreal injections of vancomycin (1 mg/0.1 ml) and amikacin (400 mcg/0.1 ml). After two unsuccessful results from aqueous and vitreous samples, pars plana vitrectomy (PPV) and iris membranectomy were performed, with additional intravitreal injections of vancomycin (1 mg/0.1 ml) and ceftazidime (2.25 mg/0.1 ml). The iris membrane was sent for microbial cultures and histopathological examination. The vitreous fluid was sent for direct microscopic examination, culture, and molecular identification of bacteria, fungi, and mycobacteria. The patient was admitted and treated with fortified vancomycin and amikacin eye drops every hour concurrent with intravenous administration of both medications (vancomycin 1 g/day and amikacin 750 mg/day). Two days after PPV, vitreous staining indicated 1+ acid-fast bacilli, but the polymerase chain reaction (PCR) was negative for mycobacteria. All other investigations for bacteria and fungi appeared negative.

**Fig. 2:**
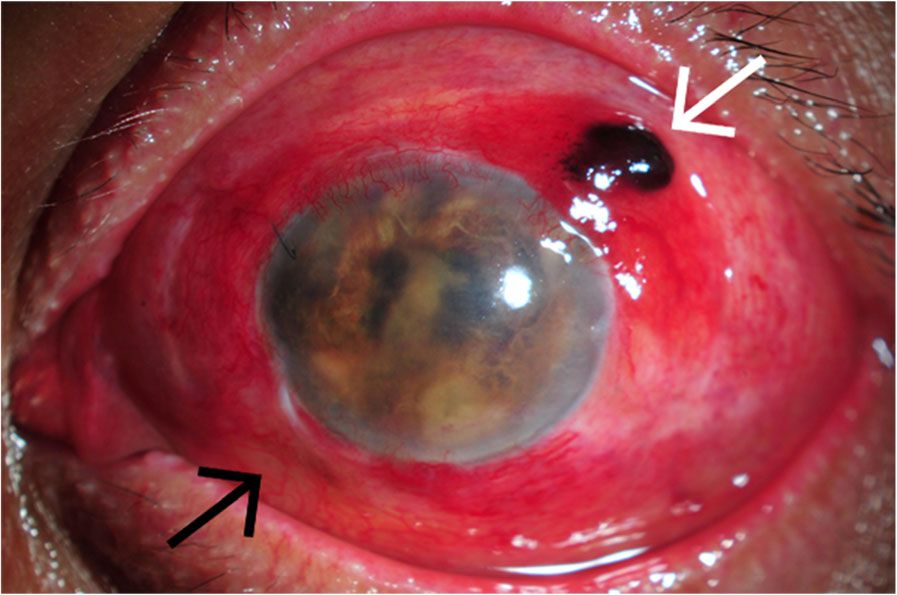
Slit lamp examination of the left eye at three months after. Prolapsed uveal tissue through a superotemporal scleral window of trabeculectomy wound (white arrow) and the inflamed inferior filtering bleb (black arrow)

According to the test results, NTM endophthalmitis was mostly suspected. The treatment was changed according to the infectious disease specialist’s recommendation to intravenous imipenem (4 g/day), levofloxacin (750 mg/day), and amikacin (750 mg/day) combined with levofloxacin eye drops every hour and tobramycin eye ointment before bedtime. After two weeks, the intravenous antibiotics were switched to oral doxycycline (200 mg/day), clarithromycin (1 g/day), and ciprofloxacin (1500 mg/day). Intermittent local injections (intravitreal, intracameral, and subconjunctival) of amikacin and imipenem were performed as an adjunctive to the systemic and topical treatments. The ocular pain and intraocular inflammation gradually improved over the treatment period. Despite the clinical improvement, the patient’s visual acuity deteriorated to no light perception (NPL), and uveal tissue had prolapsed through the superotemporal scleral window of a trabeculectomy wound (Fig. 2). Enucleation of the infected eyeball was advised to prevent sympathetic ophthalmia, but the patient declined. Three months later, vitreous cultures revealed *M. haemophilum* in liquid medium. Unfortunately, the organism could not be identified in the solid medium even after sub-culturing from liquid to solid medium, so the antibiotic susceptibility test could not be done in this patient. The final regimen was changed to oral azithromycin (500 mg/day), doxycycline (200 mg/day), and rifampicin (600 mg/day) for the next 12 months. The eye eventually became phthisical although there was no sign of recurrence or systemic infection at the five-year follow-up.

## Discussion and conclusions

To date, only three cases of *M. haemophilum* ocular infection have been published in previously reported literature [3, 10–12]. The common features were cutaneous lesions and an incomplete or impaired immune function, including young age, immunosuppressed, or uncontrolled diabetes. Cutaneous lesions were early, and were the common signs of *M. haemophilum* infection in all of the reported cases of ocular infection [3, 8, 10–12]. Therefore, ocular involvement appeared to be secondary to hematogenous spread. One of three reported cases was endophthalmitis as in our case [12]. The patient had cutaneous lesions and a gradual history of iridocyclitis before developing a suppurative, granulomatous ocular inflammation which finally required enucleation. Our patient demonstrated a similar clinical course. He had uncontrolled diabetes and developed ocular inflammation mimicking chronic granulomatous panuveitis. Subsequently, he ended up with purulent endophthalmitis and phthisis bulbi, despite the combination of intraocular and systemic antimicrobial agents.

Unlike the previously reported cases, our case was the first to demonstrate an ocular *M. haemophilum* infection without any signs of systemic association. The route of infection in our patient was assumed to be exogenous due to the history of multiple trabeculectomies. From previous studies, the five-year incidence of bleb-related endophthalmitis is 1.3%, and the common route for late post-trabeculectomy endophthalmitis is an ingress of microorganisms through the avascular bleb [13–16]. Nevertheless, in our case, the inoculation of mycobacteria through blebs could not be concluded as there was no sign of bleb inflammation at the initial presentation. Since there was no blebitis at the beginning, the condition misled us for immune-related process and thus delayed the investigations and treatment.

*M. haemophilum* infection is difficult to diagnose for the lack of specific signs, the chronic and subtle course of disease, and the special culture requirements. This slow-growing mycobacterium usually takes up to eight weeks to grow in a medium in the temperature range of 30-32 °C, which is lower than that required by other NTMs (35–37 °C). Specifically, it needs iron supplementation in the culture medium and this characteristic gives it the name “haemophilum.” Improper culture techniques may lead to a false-negative result [3, 8, 11, 17, 18]. A regimen for *M. haemophilum* diagnosis includes acid-fast stain with Ziehl-Neelsen techniques and two culture conditions: 1) standard protocol, using Mycobacteria Growth Indicator Tube (MGIT) and Löwenstein-Jensen (LJ) medium at 35 °C; and 2) *M. haemophilum*-specific protocol, using LJ medium with iron supplementation at 30 °C. Molecular detection is also recommended because a PCR assay provides higher sensitivity for *M. haemophilum* detection [3]. In our case, although treatment was initiated as soon as NTM was suspected, it took three months to identify the organism.

Even though the drug susceptibility of microorganism in our case was not established, previous studies revealed that NTM was commonly susceptible to antituberculosis, fluoroquinolones, macrolides, and aminoglycosides [3, 14, 20]. Therefore, our suggested treatment is a long-term systemic combination of rifampicin, ciprofloxacin, doxycycline, and azithromycin. Additional intravitreal injection of amikacin was also recommended for NTM intraocular infection [3, 14]. However, specific guidelines for the antibiotics, the routes of administration and the duration of treatment have not been developed [3, 8, 19].

The prognosis depends on early diagnosis, appropriate therapy, and immune status of the patient which affects immune function [20]. White et al. described that early diagnosis could reduce morbidity and mortality in bone marrow transplant recipients with *M. haemophilum* infection [21]. Fairhurst et al. emphasized the importance of early treatment which led to good outcome, especially in disseminated *M. haemophilum* infection [22]. Similar to the previous report, our case culminated with NPL and phthisis bulbi, despite aggressive treatments with local and systemic antimicrobial agents. This should alert the ophthalmologist to perform microorganism identification including NTM as soon as infection is suspicious.

Although rare, NTM endophthalmitis can be devastating to the eye. Unlike previous reports, our case was the first of exogenous endophthalmitis being caused by *M. haemophilum*. This case signals the need to be aware of NTM infections when face with cases of chronic-onset granulomatous intraocular inflammation, and with histories of previous intraocular intervention, immunocompromised status, and/or corticosteroid resistance. Early identification of the microorganisms and proper treatments are required to minimize the risk of losing a patient’s eye. However, guidelines for investigation and therapeutic intervention are not well established.

## Abbreviations

BCVA: best-corrected visual acuity
HM: hand motion
IOP: intraocular pressure
KPs: keratic precipitates
LJ: Löwenstein-Jensen
*M. haemophilum*: *Mycobacterium haemophilum*
MGIT: Mycobacteria Growth Indicator Tube
NIDDM: non-insulin-dependent diabetes mellitus
NPL: no light perception
NTM: Non-tuberculous mycobacteria
PCR: polymerase chain reaction
PJ: light projection
POAG: primary open angle glaucoma
PPV: pars plana vitrectomy
